# Directed Mutational Strategies Reveal Drug Binding and Transport by the MDR Transporters of *Candida albicans*

**DOI:** 10.3390/jof7020068

**Published:** 2021-01-20

**Authors:** Atanu Banerjee, Jorgaq Pata, Suman Sharma, Brian C. Monk, Pierre Falson, Rajendra Prasad

**Affiliations:** 1 Amity Institute of Biotechnology, Amity University Haryana, Gurgaon 122413, India; abanerjee1@ggn.amity.edu (A.B.); sssumansharma21@gmail.com (S.S.); 2 Drug Resistance & Membrane Proteins Team, Molecular Microbiology and Structural Biochemistry Laboratory, Institut de Biologie et Chimie des Protéines, CNRS-Lyon 1 University UMR5086, 69367 Lyon, France; pata.jorgaq@ibcp.fr; 3 Sir John Walsh Research Institute, Faculty of Dentistry, University of Otago, Dunedin 9016, New Zealand; brian.monk@otago.ac.nz; 4 Amity Institute of Integrative Sciences and Health, Amity University Haryana, Gurgaon 122413, India

**Keywords:** *Candida albicans*, ABC transporters, MFS transporters, multidrug efflux pumps, *Ca*Cdr1, *Ca*Mdr1, polyspecificity, interdomain crosstalk, mutagenesis, suppressor genetics

## Abstract

Multidrug resistance (MDR) transporters belonging to either the ATP-Binding Cassette (ABC) or Major Facilitator Superfamily (MFS) groups are major determinants of clinical drug resistance in fungi. The overproduction of these proteins enables the extrusion of incoming drugs at rates that prevent lethal effects. The promiscuity of these proteins is intriguing because they export a wide range of structurally unrelated molecules. Research in the last two decades has used multiple approaches to dissect the molecular basis of the polyspecificity of multidrug transporters. With large numbers of drug transporters potentially involved in clinical drug resistance in pathogenic yeasts, this review focuses on the drug transporters of the important pathogen *Candida albicans*. This organism harbors many such proteins, several of which have been shown to actively export antifungal drugs. Of these, the ABC protein *Ca*Cdr1 and the MFS protein *Ca*Mdr1 are the two most prominent and have thus been subjected to intense site-directed mutagenesis and suppressor genetics-based analysis. Numerous results point to a common theme underlying the strategy of promiscuity adopted by both *Ca*Cdr1 and *Ca*Mdr1. This review summarizes the body of research that has provided insight into how multidrug transporters function and deliver their remarkable polyspecificity.

## 1. Introduction

Human pathogenic yeast that include *Candida albicans* and non-*albicans Candida* (NAC) species are commensal pathogens infecting individuals with compromised immunity [1]. The superficial infections caused by *C. albicans* and NAC species can also extend to disseminated bloodstream and deep-tissue infections [1]. Although C. *albicans* and a few NAC species (e.g., *Candida glabrata*, *Candida tropicalis*, and *Candida parapsilosis*) are considered to be the most common fungi infecting immunocompromised patients, more recently, *Candida auris* has been recognized as a global health threat (https://www.cdc.gov/fungal/candida-auris/index.html) [2]. Besides the ability to spread nosocomially, its propensity to form adherent biofilms on medically relevant substrates has led to numerous hospital outbreaks of *C. auris* globally [3]. A higher percentage of clinical isolates resistant to multiple classes of antifungal agents is the greatest challenge posed by this recently emerged NAC species [4]. Apart from *C. auris*, the prophylactic or, prolonged use of antifungal drugs has allowed many other *Candida* species to manifest resistance to azoles, polyenes, echinocandins, and pyrimidine analogues [4,5]. Compared to other classes of antifungals, resistance to azole antifungals is much more common, presumably due to their fungistatic nature.

*C. albicans* and NAC species have used many strategies to deal with the onslaught of common antifungals [6]. One of the most prominent of these mechanisms is the ability of *Candida* species to rapidly efflux incoming drugs [6]. This feature is helped by a group of drug transporters belonging to the ATP-Binding Cassette (ABC) and Major Facilitator Superfamily (MFS) classes of proteins [7]. *Candida* cells harbor a battery of both ABC and MFS proteins; however, only a few have a well-established role in clinical drug resistance. While the ABC proteins are primary transporters that couple ATP-binding and hydrolysis to power drug extrusion, MFS transporters are secondary transporters that instead exploit the electrochemical gradient of protons to facilitate drug efflux [7]. Both ABC and MFS proteins are promiscuous transporters with the ability to export a diversity of substrates across membranes. Among the prominent transporters that have a proven role in azole resistance, the ABC proteins *Candida albicans* drug resistance protein 1 (*Ca*Cdr1) and *Candida albicans* drug resistance protein 2 (*Ca*Cdr2), and the MFS protein *Candida albicans* multidrug resistance protein 1 (*Ca*Mdr1) stand out in terms of their clinical relevance [6,7,8]. Most azole-resistant clinical isolates show overexpression of genes encoding these ABC and MFS proteins [6,9,10,11]. The rapid efflux of incoming drugs by these transporters prevents the retention of the drugs at detrimental concentrations, thus facilitating cell survival. These drug efflux proteins appear to undergo substantial conformational change during drug transport [7,12]. How ranges of diverse substrates are bound and transported are some of the questions that have been addressed significantly in several recent studies. The focus of this article is to highlight recent advances in the knowledge of transporters belonging to *C. albicans* and track landmark research that has helped elucidate structural and functional understanding of these proteins.

## 2. Historical Background of the MDR Pumps in Yeast

ABC proteins were first identified in bacteria as prominent nutrient importers and came to center stage when their homologues were shown to cause multidrug resistance in cancer cells [13,14,15]. In yeast, Rank and Bech-Hansen identified a point mutation in a gene (later named Pleiotropic drug resistance 1 (*PDR1*)) that led to increased xenobiotic resistance [16]. Goffeau’s group subsequently established that it conferred resistance to a number of antifungal drugs including ketoconazole and cycloheximide [17]. Since then, numerous additional *PDR1* gain-of-function mutations have been reported [18]. Golin’s group identified the first target of *PDR1* from a genomic DNA library. It was found within a DNA fragment that conferred resistance to cycloheximide and sulfometuron methyl and was named the Pleiotropic drug resistance 5 (*PDR5*) gene [19]. Myers and colleagues then demonstrated that deletion of the *PDR5* gene led to marked hyper-susceptibility to a number of antifungal compounds and some in vitro inhibitors including chloramphenicol [20]. Goffeau’s group demonstrated that the gene belonged to the ABC superfamily and had the ability to transport a number of molecules including some anticancer drugs and rhodamines [21]. A *PDR5*-like gene was soon identified by a functional complementation of *PDR5* using a *C. albicans* genomic library. Sequencing of the complementing genomic fragment revealed an open reading frame (ORF) with close homology with *PDR5*, and was designated as *Candida* Drug Resistance 1 gene (*CDR1*) [22]. This was a turning point as *CDR1* was soon established as one of the major determinants of antifungal resistance in *C. albicans* [10]. *CaCDR1* identification quickly led to the identification of other homologues such as *CaCDR2*, *CaCDR3*, and *CaCDR4*. Of these, only *Ca*CDR2 was shown to play a role, albeit minor, in antifungal resistance due to its export of antifungals including azole drugs [23,24,25,26].

Based on sequence similarity, the ABC proteins in all organisms are divided into nine subfamilies, from ABCA to ABCI, according to the Human Genome Organization (HUGO) nomenclature [27]. An initial inventory by Gaur et al. of *C. albicans* ABC proteins contained 28 putative members [28]. Subsequent modifications in the genome assembly found 26 members that can be clustered into six subfamilies designated ABCB/MDR, ABCC/MRP, ABCD/ALDP, ABCF/YEF3, ABCE/RLI, and ABCG/PDR [6]. Since the members of ABCB/MDR, ABCC/MRP, ABCD/ALDP, and ABCG/PDR possess transmembrane domains (TMDs), they are putative membrane-localized transporters. The ABCF/YEF3 and ABCE/RLI representatives lack transmembrane components and have been shown to participate in non-transport functions such as translation initiation and regulation, ribosome biogenesis, etc. [6,29]. The ABCG/PDR subfamily is the largest among all *Candida* species: 9 among 26 in *C. albicans* [6], 7 among 25 in *C. glabrata* [30], and 7 among 28 in *C. auris* [31]. Characterization of the four PDR subfamily members in *C. albicans* (*CaCDR1-4*) showed that only *CDR1* and *CDR2* encode drug and phospholipid transporters. *CDR3* and *CDR4* do not encode drug transporters but instead translocate phosphoglycerides between the two lipid monolayers of plasma membrane [32,33,34].

The MFS superfamily is a vast family of transporters that is ubiquitous in the Kingdom of Life. Its members function as uniporters, antiporters, and symporters for a wide range of substrates from nutrients to drugs [35,36]. Yeast, including *Candida* species, are no exception and harbor large numbers of MFS proteins [37]. While the role of MFS proteins as transporters was well recognized in bacteria, the first realization of MFS protein involvement in drug resistance came when Fling et al. identified the *C. albicans* MFS transporter encoding the gene designated *BEN^r^* (for benomyl resistance) [38]. It conferred resistance to benomyl and methotrexate in a susceptible *Saccharomyces cerevisiae* strain and had similarity to genes encoding antibiotic resistance in prokaryotes and eukaryotes. This included a high degree of identity to the cycloheximide resistance gene in *C. maltosa* [39]. *BEN^r^* was also shown to confer resistance to many structurally and functionally unrelated compounds including cycloheximide, benzotriazoles, 4-nitroquinoline-N-oxide, and sulfometuron methyl [40]. As increased Ben*^r^* levels conferred resistance to diverse substrates and thus functioned as a multidrug transporter, its gene was redesignated as *CaMDR1* [39]. Subsequent research identified *CaMDR1* homologues in other *Candida* species.

MFS proteins typically consist of 400–600 amino acids and analysis of their primary sequences revealed that within each family, sequence similarity is highly significant [41]. In the *S. cerevisiae* genome, sequences encoding a total of 22 MFS proteins have been identified belonging to either Drug:H^+^ Antiporter family 1 (DHA1) or Drug:H^+^ Antiporter family 2 (DHA2), which differ in number of transmembrane helices (TMHs) [41]. Members of the DHA1 family have 12 TMHs, while DHA2 members possess 14 TMHs. Bioinformatics analysis of *C. albicans* MFS proteins identified 95 members in 17 families, with DHA1 and DHA2 as the major families, comprising of 22 and 9 representatives, respectively [37]. The well characterized MFS drug transporter *Ca*Mdr1 belongs to the DHA1 family [41].

Despite the large number of PDR subfamily and DHA1 family members within the ABC and MFS superfamilies, respectively, only *Ca*Cdr1, *Ca*Cdr2, and *Ca*Mdr1 have demonstrated clinical significance as multidrug transporters [42]. What structural features enable this select group of proteins to be promiscuous transporters that are able to bind and release ranges of unrelated xenobiotics? Researchers have addressed such questions for over two decades. The following sections highlight various strategies used to probe the mechanisms of the two major multidrug transporters of *C. albicans*—*Ca*Cdr1 and *Ca*Mdr1. For clarity, the strategies employed to analyze structural and functional aspects are discussed separately.

## 3. Structural Organization of *Ca*Cdr1 and *Ca*Mdr1

### 3.1. CaCdr1

**Figure 1:**
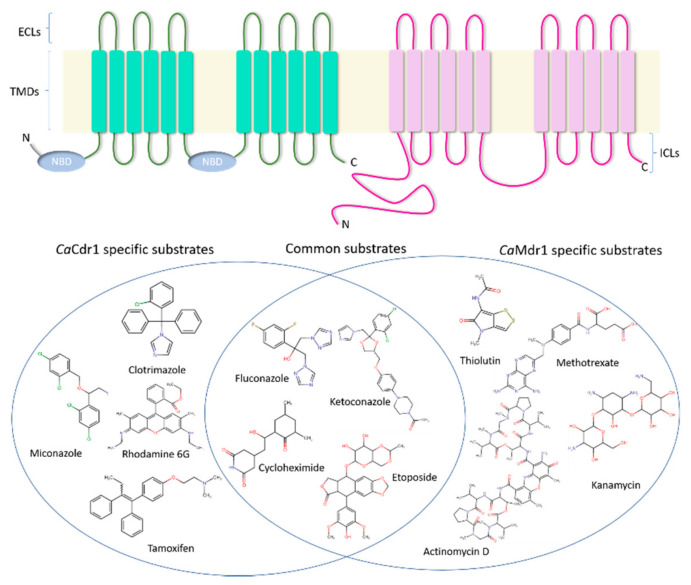
General topology of the two major MDR pumps of *Candida albicans*, *Ca*Cdr1 and *Ca*Mdr1, and their substrate promiscuity. *Ca*Cdr1 is made up of two transmembrane domains (TMDs) and two nucleotide binding domains (NBDs) arranged in a reverse topology. Each TMD is made up of six transmembrane helices (TMHs) which are connected by four intracellular loops (ICLs) and six extracellular loops (ECLs). *Ca*Mdr1 comprises two TMDs, each made of six TMHs connected by five ICLs (the third ICL is the largest and also referred to as the central cytoplasmic loop (CCL)). In addition, it also contains a large N-terminal extension. The Venn diagram in the lower panel represents the common and specific substrates of these pumps. The source of this information is the study by Nidhi et al. [46]. The structures are drawn using the Chemaxon tool.

All functional ABC transporters are essentially built upon a similar structural topology that includes two transmembrane domains (TMDs) and two nucleotide-binding domains (NBDs) [43]. These four domains can be present as part of a single polypeptide as in the case of full transporters like *Ca*Cdr1 or as two separate polypeptide units each comprising one TMD and one NBD (half transporters) [27]. In the forward topology, the TMD precedes the NBD (TMD-NBD), whereas NBD comes first in the case of reverse topology (NBD-TMD), the latter being the feature of *Ca*Cdr1 [27]. Functionally, while NBDs bind and hydrolyze ATP to power drug efflux, TMDs are the substrate recognition entities [6]. Interestingly, the NBDs contain a number of highly conserved hallmark motifs that aid in the various processes of ATP binding and hydrolysis and communication with other domains [44]. *Ca*Cdr1 is a 169.9 kDa protein built on the structural plan of a full ABC transporter but with its two homologous halves in reverse topology [27]. Each TMD is made up of six transmembrane helices (TMHs) [6]. The TMHs are interlinked by six extracellular loops (ECL1–6) and four intracellular loops (ICL1-4) [6,45] (Figure 1). The NBDs have the hallmark β-sheet sub-domain containing the Walker A and Walker B motifs and an α-helical sub-domain that consists of the ABC signature sequence [27].

*Ca*Cdr1 and other PDR transporters have special features compared with other members of the ABC superfamily. PDR transporters have a constitutively active ATPase machinery which is not dependent on substrate exposure [47]. Furthermore, they do not show significant drug-stimulated ATPase activity, in contrast with other well-known ABC pumps such as the P-glycoprotein [48]. At the level of primary structure, *Ca*Cdr1 and its *S. cerevisiae* homolog *Sc*Pdr5 display many atypical substitutions within the otherwise well conserved motifs of the NBDs [6,49]. These substitutions are highlighted in Table 1. As a result of these substitutions, only one of the two nucleotide-binding sites (NBS) contains the canonical motifs. The site containing the canonical motifs is usually referred to as the canonical NBS, while the other that comprises atypical substitutions is denoted as the non-canonical/deviant/atypical NBS [50]. It is important to note that asymmetry at the level of NBS is not restricted to the PDR pumps. It is also seen in some other clinically relevant ABC pumps including the Cystic Fibrosis Transmembrane Conductance Regulator (CFTR) and the Transporter associated with antigen processing (TAP1/TAP2) complex [51,52]. Several attempts have been made to understand the importance of the asymmetry within the NBDs of these pumps. Interestingly, most studies suggest a non-catalytic role for the non-canonical NBS of transporters like CFTR and TAP1/TAP2 [53,54]. The canonical NBS of PDR pumps like *Sc*Pdr5 and *Ca*Cdr1 appears to have a functional role [50,55,56,57,58]. Studies of *Ca*Cdr1 pointing towards such a role are highlighted in a later section.

Differences specifically associated with PDR transporters are seen at the level of the extracellular domains which contains two very long ECLs, ECL3, and the partially homologous ECL6 [45]. In contrast, the TMDs are poorly conserved, as is the case for most ABC pumps. This primary sequence variation is probably the main reason for the polyspecificity of these pumps. Interestingly, despite the variation in the *Ca*Cdr1 TMDs, significant conservation was found in select TMHs by Rawal et al. [59]. Conservation scores among all the TMHs showed TMH2 to be the most conserved, while TMH10 was the least conserved. However, no direct correlation was found between conservation scores and functional importance within the transport mechanism [59]. The structure–function studies used to obtain insight into the substrate promiscuity of *Ca*Cdr1 manifested by the TMDs are described in a subsequent section.

### 3.2. CaMdr1

*Ca*Mdr1 is a typical DHA1 family antiporter containing 12 TMHs connected by hydrophilic loops, with both the N- and C-termini located in the cytoplasm [41] (Figure 1). In total, there are five ICLs and six ECLs in *Ca*Mdr1. Interestingly, the N-terminal half of the protein (TMH1 to TMH6) shows weak sequence homology to the C-terminal half (TMH7 to TMH12) [41]. Similar to other DHA1 subfamily members, *Ca*Mdr1 contains the “antiporter motif” [^244^G(X)_6_G(X)_3_GP(X)_2_GP(X)_2_G^263^] within TMH5 [60]. Additional unique features of the *Ca*Mdr1 include: (1) the presence of a long hydrophilic N-terminal extension, and (2) a large ICL3, often referred to as the central cytoplasmic loop or CCL, which is dominated by non-polar amino acids within its helical regions [60,61]. Interestingly, sequence alignment of the *Ca*Mdr1 CCL with that of the other DHA1 representatives did not reveal significant conservation [61]. Nonetheless, the helix propensities and hydrophobicity of the CCL of all fungal DHA1 proteins were of similar order. Deletion and mutational studies by Mandal and colleagues demonstrated its importance in the maintenance of protein conformation and substrate transport [61]. However, the importance of the N-terminal extension in *Ca*Mdr1 is yet to be elucidated.

## 4. Insights into the Drug Binding Pocket of *Ca*Cdr1 and *Ca*Mdr1

As both *Ca*Cdr1 and *Ca*Mdr1 are membrane proteins, studying the properties of purified enzymes has been inherently difficult. In order to achieve the in-depth characterization of these pumps within their native environment, an effective overexpression system that offered minimum background from other membrane-bound transporters was required. This demand was met by the heterologous *S. cerevisiae* AD1-8u^−^ system developed by Decottignies et al. in Goffeau’s group and later augmented with expression vectors and homologous recombination approaches developed by the Monk and Cannon group for high-level constitutive expression of tagged versions of these proteins [62,63]. A primary reason for the success of the AD1-8u^−^ host system and its derivatives is because it is depleted of the seven endogenous drug efflux pumps (Pdr5, Yor1, Snq2, Ycf1, Pdr10, Pdr11, and Pdr15) and hence, has limited intrinsic drug efflux capability. Furthermore, the strain harbors a gain of function mutation in the *PDR1* regulator (pdr1–3 mutation) controlling the *PDR5* promoter utilized for target protein overexpression. Overall, this system facilitates high-level constitutive expression of the target protein with minimum background from other drug efflux pumps. The system has been extensively utilized to study the yeast drug transporters *Ca*Cdr1 and *Ca*Mdr1.

### 4.1. CaCdr1

Use of the AD1-8u^−^ host system helped address the importance of specific TMHs in substrate binding and transport by *Ca*Cdr1. For example, Puri et al. and Saini et al. employed site-directed mutagenesis to reveal that TMH5 and TMH11 harbor significant numbers of residues which participate in drug recognition and transport [64,65]. In order to understand the molecular basis of polyspecificty in *Ca*Cdr1, Rawal et al. used a more extensive directed mutational analysis to map and dissect the nature of the drug binding cavity [59]. The entire primary sequence of the 12 TMHs was subjected to systematic mutagenesis with each amino acid residue within the 12 TMHs replaced with an alanine (alanines were replaced with glycine). The resulting library of 252 mutant variants gave opportunity to probe the role of each TMH residue in the drug transport cycle. These variant proteins overexpressed in the AD1-8u^−^ host system were assessed for their drug binding, drug transport, and ATP hydrolysis capabilities. This extensive phenotypic, biochemical, and biophysical characterization revealed that the individual replacements of about 70% of the native residues with alanine (or glycine, as required) gave native or wild type (WT) phenotypes. In contrast, substitution of approximately 30% of the residues yielded *Ca*Cdr1 variants that were completely, or selectively, drug sensitive, albeit showing proper surface localization like the WT. Thus, it was possible to segregate critical residues in the TMDs from the neutral ones. Major insight was obtained by using the ability to identify lipid bilayer-facing surfaces on each TMH using the empirical scoring function LIPS (Lipid-facing Surface). Despite the majority of the critical residues being hydrophobic in nature, with few exceptions, these residues were also non-LIPS (not facing the lipid environment) [59]. The study not only found clustering of deleterious and neutral mutations in the TMHs but also pointed out that many non-LIPS residues may play significant structural or functional roles in drug recognition and transport, even if not directly contributing to the proposed drug binding site between the two TMDs. A further key finding was that residue conservation score per se does not directly translate into its importance in substrate binding and/or transport. By considering the relevance of individual TMHs, it was found that the majority of residues participating in the formation of substrate binding site(s) belonged to TMH1, TMH2, TMH4, TMH5, TMH8, and TMH11 [59]. This study was conducted at a time when structures from the ABCG subfamily (PDR subfamily in the case of yeasts) were not available, and hence, modeling was restricted only to the available templates from other subfamilies. With the availability of ABCG structures, particularly ABCG5/G8 from Lee et al., it became evident that the ABCG transporters adopt a distinct conformation, known as the type II fold of ABC exporters [66], more recently re-baptized type V in a more global classification [67]. Distinctive features include the absence of crossover between the homologous halves of the transporter and the existence of a large but narrow substrate-binding cavity [66]. A subsequent ABCG2 structure further reinforced these insights [68].

By exploiting the *Ca*Cdr1 TMH substitution library, Nim et al. screened three families of structurally related compounds (i.e., rhodamines, tetrazoliums, and tin chlorides) to dissect their interactions with *Ca*Cdr1 [69]. Rhodamines are highly preferred substrates of PDR pumps and are routinely used in substrate transport assays. Since the rhodamines are of a similar size but differ in the overall charge (i.e., Rhodamine 6G (R6G) and Rhodamine 123 (R123) are positively charged but Rhodamine B (RB) is negatively charged), the screen was able to assess the importance of substrate charge. The panel of rhodamines when screened against the *Ca*Cdr1 TMH alanine substitution library provided several valuable insights. First, only 20% of the TMH residues were found to be critical for rhodamine transport and the substitutions of the remaining 80% residues did not alter susceptibility towards rhodamines [69]. Secondly, substrate charge emerged as a feature that affects interactivity with *Ca*Cdr1. Out of the 252 total residues, only 3% appeared critical in the case of negatively charged RB as compared to 13% for the positively charged R6G and R123 [69]. Moreover, 5% of the residues were found to give common phenotypes in response to both the negative and positively charged substrates. Positively charged rhodamines were thus concluded to be the preferred substrates for *Ca*Cdr1. The study also identified new substrates of *Ca*Cdr1 including tetrazolium chloride and trimethyltin chloride [69]. A more incisive structural view of the substrate binding site(s) was provided when the ABCG5/G8 structure had become available as a template for *Ca*Cdr1 modeling [69]. In the case of rhodamines and tetrazoliums, their binding site primarily involves the N-terminal half of the pump, between TMH 2 and TMH11, and surrounded by TMH1 and TMH5 [69]. In contrast, tin chlorides engage both the N- and C-terminal halves of *Ca*Cdr1, binding at the interface of TMH 2, 11, 1, and 5 [69]. The screen also identified for the first time the involvement of TMH12 residues in effluxing tetrazolium chloride, trimethyltin chloride, and the Ca^2+^ ionophore A23187 [69].

Baghel et al. used the same library to investigate the importance of individual TMHs in β-estradiol and corticosterone transport [70]. Surprisingly, they found that a major proportion of the residues affecting sterol binding (54% and 83% for β-estradiol and corticosterone, respectively) face the lipid interface [70]. Molecular modeling suggested a peripheral corticosterone binding site between TMHs 3, 4, and 6 and with β-estradiol site engaging TMHs 2, 5, and 8. Some residues critical for β-estradiol efflux overlap with those required for R6G transport, a function with which β-estradiol competes [69,70].

### 4.2. CaMdr1

As noted above, all DHA1 family member transporters harbor a typical antiporter motif known as motif-C. An initial mutational study by Pasrija et al. aimed to evaluate the functional relevance of motif-C (within TMH5) in *Ca*Mdr1 drug transport mechanism [60]. Replacement of the first four glycines (i.e., G244G, G251, G255, and G259) within the “G244(X)_6_G(X)_3_GP(X)_2_GP(X)_2_G263” motif with alanine yielded protein variants deficient in transport capabilities. In contrast, the G263A mutation did not interfere with CaMdr1 localization or its transport attributes [60]. Alanine substitution of some of the other residues within the motif led to protein variants that had transport defects and consequently failed to confer resistance towards drug substrates [60]. To investigate the TMH residues in more detail, Kapoor et al. employed the information theory methods—relative entropy (RE_M_) and cumulative relative entropy (CRE) [71,72]. RE_M_ and CRE scoring identified fold-specific and function-specific residues from multiple sequence alignments. Site-directed mutagenesis of high RE_M_ residues within *Ca*Mdr1 demonstrated their importance in the maintenance of structure and transport function [71]. It was also found that important residues were not just restricted to the conserved motifs. A second study from the same group used CRE scoring to compare the DHA1 (antiporters) and Sugar Porter (SP; symporters) family representatives in order to identify residues that impart substrate specificity upon *Ca*Mdr1 [72]. Site-directed mutagenesis of high scoring residues revealed their importance in conferring complete or selective sensitivity towards the drug substrates tested [72]. Rational mutagenesis by Kapoor et al. validated data from previous studies, wherein aligned residues in other members of the DHA1 family were also found to be functionally critical [72]. Both studies demonstrated the utility of information theory measures in evaluating the functional significance of different amino acid residues in transporter proteins.

Since *Ca*Mdr1 utilizes proton-motive force to power substrate efflux, a system to monitor proton influx coupled to substrate transport was required. Redhu et al. modified the AD1-8u^−^ system to effectively monitor the import of protons as well [73]. This was achieved by using the ratiometric pH-sensitive GFP probe pHluorin to measure cytosolic pH and its correlation with drug efflux for each of the charged residues within the transmembrane domains of *Ca*Mdr1 [73]. Mutagenesis and biochemical analyses identified R215 within the TMH4 as a major determinant of the substrate:H^+^ antiport mechanism in *Ca*Mdr1 [73].

**Figure 2:**
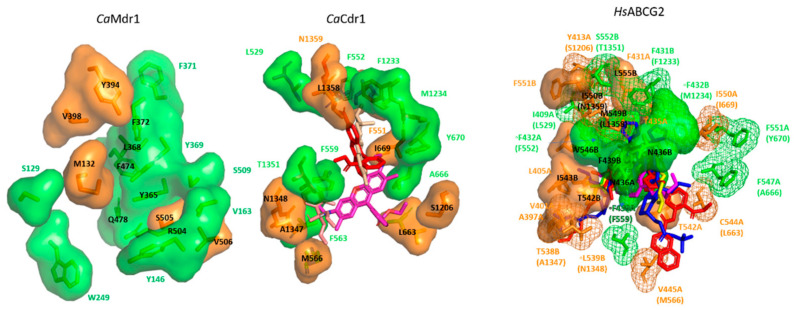
Structural basis for polyspecificity of multidrug efflux pumps. Left panel, GlpT-based model of *Ca*Mdr1 amino acid residues of interest [74]. Middle panel: ABCG5-G8-based model of *Ca*Cdr1 amino acid residues of interest with Rhodamine 6G (magenta), fluconazole (light pink), and rhodamine 123 (red) docked [69]. Right panel: cryo-electron microscopy structures of human ABCG2 in complex with substrates and inhibitors, PDB codes: 6FEQ [75], 6FFC [75], 6HCO [76], 6VXI [77], 6VXJ [77], 6VXH [77]. Residues are shown on the surface, with those always implicated in substrate binding in green and those involved in binding specific ligands (polyspecificity) in orange. Residues shown in the mesh in the right panel correspond to similar residues of *Ca*Cdr1, indicated in brackets.

Similar to the study on *Ca*Cdr1, Redhu et al. performed alanine (glycine substitution in places where alanine is present) scanning mutagenesis for all the residues present within the TMDs of *Ca*Mdr1 [74]. The library of 252 mutants (created by overexpressing all the alanine or glycine variants in the AD1-8u^−^ host strain) was screened against different drug substrates of *Ca*Mdr1 [74]. The results showed that around 33% (84/252) of the variants with alanine (or glycine) substitutions gave total or selective loss of drug resistance [74]. Of these 84 variants, 53 were sensitive to all the tested drugs, and the remaining 31 were sensitive to at least one [74]. Notably, only 5 out of the 84 drug susceptible variants showed poor cell surface expression and/or partial trapping of the protein within the intracellular structures [74]. Conservation analysis showed that TMH4 is maximally conserved among all the TMHs and contains the greatest number of critical residues. These observations contrasted starkly with *Ca*Cdr1, where there was no direct correlation between conservation scores of the TMHs and their relative importance [59]. However, apart from *Ca*Mdr1 TMH4, there was no direct relationship between the TMH conservation score and functional importance [74]. Importantly, this study not only provided a comprehensive functional classification of critical residues but also a view of their putative arrangements within the TMDs. This was achieved by exploiting as a template a GlpT-based 3D model of *Ca*Mdr1 and inputs from biochemical analyses [74]. The residue classes included (i) ones with a structural impact (“S” group), (ii) residues facing the lipid interface (“L” group), (iii) residues buried but not facing the main central pocket (important for substrate:H^+^ antiport mechanism, “M” group), and (iv) residues that are buried and face the main central pocket (“B” group). Within the “B” category, there are five residues that are substrate-specific (“P” group) (Figure 2). Structural superimposition of the *Ca*Mdr1 model with substrate-specific MFS pumps like Glut1 and XylE, and polyspecific pumps like MdfA implied that the “B” group forms a central binding pocket, and the P group surrounds the B core [74]. These observations suggested that the polyspecificity of MFS drug transporters involves residues situated at the periphery of the central substrate binding core that enable the accommodation of compounds differing in size and shape. [74].

If true, this distribution of core/peripheral residues could also be observed in other MDR pumps such as *Ca*Cdr1. Data obtained with the *Ca*Cdr1 TMH substitution mutant library described above were therefore re-examined following the same scheme used with *Ca*Mdr1 (Figure 2). Among the 74 *Ca*Cdr1 residues critical for substrate transport, 25 were found to belong to the “Mechanism group”, 9 to the “Binding group”, and 24 to the “Lipid group”. Five residues were found in the “Structure group”. These were mainly positioned at the top of the cavity and were small residues (A660, G672, G1366, A1346, G1333, A1286) that may be important for the structural flexibility of the binding cavity. Finally, seven residues were found to belong to the Polyspecificity group. The position of these residues in the 3D model of *Ca*Cdr1 indeed revealed a drug-binding cavity made of a main binding core surrounded by three peripheral “P” zones, in a pattern like that found in *Ca*Mdr1. This finding strengthens the view that the polyspecificity in multidrug efflux pumps lies in an extended capacity for ligand-binding brought about by few residues arrayed at the periphery of a core-binding space. The substrate-binding pocket of *Ca*Cdr1 is reminiscent of human ABCG2, which shares the same reverse topology of the G-subfamily proteins and whose 3D structure has been determined with various ligands [75,76,77]. The substrate binds in a slit-like pocket at the crevice between the TMDs within strongly overlapping pockets, but with additional contacts depending on the nature of the substrate (Figure 2).

Both *Ca*Cdr1 and *Ca*Mdr1 have broad promiscuity for a range of structurally and functionally distinct substrates. Besides antifungal drugs, a host of other molecules including fluorescent probes, herbicides, lipid species, and protein synthesis inhibitors are exported [6,41,46]. Figure 1 illustrates the substrate promiscuity of *Ca*Cdr1 and *Ca*Mdr1 and highlights the overlap in their substrate ranges. One initial Structure–Activity Relationship (SAR) study attempted to deduce the features that tend to distinguish substrates of *Ca*Cdr1 and *Ca*Mdr1 [46]. It highlighted features displayed by *Ca*Cdr1 substrates but was unable to deduce a conclusive set of features for the *Ca*Mdr1 substrates. The SAR analysis of Puri et al. showed that high hydrophobicity index, presence of an atom-centered fragment (R–CH–R), and molecular branching are the most common features presented by *Ca*Cdr1 substrates [46]. The TMD mutagenesis studies carried out with *Ca*Cdr1 and *Ca*Mdr1, plus the further analysis highlighted above, have shed light on the molecular basis of substrate promiscuity in these pumps. In both cases, a central binding pocket formed by certain helices of the TMDs is augmented by certain residues situated at the periphery of the central core in order to confer polyspecificity [59,74].

## 5. Interdomain Crosstalk in *Ca*Cdr1 and *Ca*Mdr1

Among the PDR transporters, Golin’s group made early attempts to investigate the interdomain crosstalk within the Pdr5 protein of *S. cerevisiae*. They first identified the S558Y mutation within the membrane helix 2 that rendered the yeast cells susceptible to the antifungal substrate cycloheximide [78]. This mutation, however, did not alter basal ATPase activity. The group then exploited a suppressor genetics strategy because the mutant phenotype suggested a defect in crosstalk across NBDs and TMDs. Interestingly, amongst all the suppressor mutations that alleviated the S558Y transport defect, a significant number was found close to or within the Q-loop of the non-catalytic NBS [79]. These results led the authors to propose a plausible role of the Q-loop in interdomain crosstalk [79].

**Figure 3:**
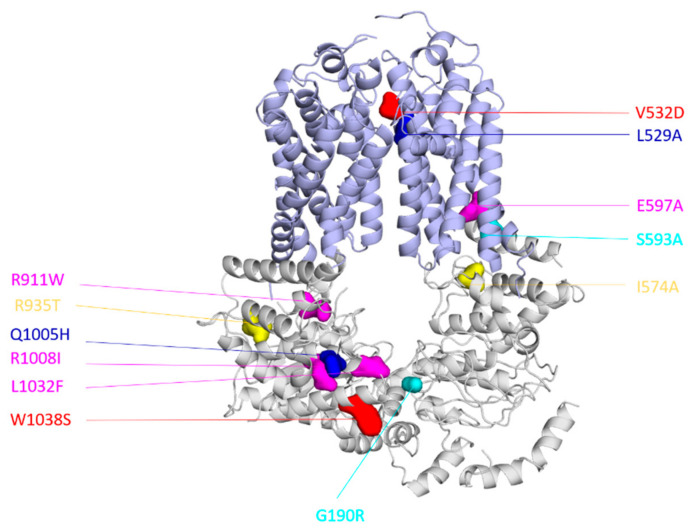
Interdomain crosstalk identified in *Ca*Cdr1 through suppressor genetics. Cartoon representation of a 3D model of *Ca*Cdr1 highlighting the primary mutants within the TMHs and ICLs and their recovered suppressors. In red: the mutant V532D and its suppressor W1038S [58]. In blue: the mutant L529A and its suppressor Q1005H [50]. In magenta: the mutant E597A and its suppressors R911W, R1008I, and L1032F [80]. In yellow: the mutant I574A and its suppressor R935T [80]. In cyan: the mutant S593A and its suppressor G190R [80].

In the case of *Ca*Cdr1, exploring the interdomain crosstalk started with a study by Shah et al. of the ICLs [80]. Because the ICLs provide a signaling interface between the TMDs and NBDs [81], all the amino acids within the ICLs were tested for their ability to impact transport profiles and ATP hydrolysis [80]. Alanine scanning mutagenesis was performed for all the amino acids within the four ICLs and the resultant overexpression library of the 84 mutants subjected to multiple phenotypic mapping and biochemical analyses [80]. Only 18% of the mutants showed an alteration in drug resistance profile and the remaining mutants had neutral phenotypes [80]. Notably, most of the critical mutants belonged to ICL1. With the exception of a few mutants, most drug susceptible ICL mutants involve replacement of the non-conserved residues within the ICLs [80]. Furthermore, all the mutants that were susceptible to triazoles were also susceptible to imidazoles, but not vice versa, indicating selectivity. In line with these expectations, ATPase activity was uncoupled from substrate transport in most of the affected mutants [80]. To evaluate the interdomain crosstalk, some of the most critical alanine variants were subjected to suppressor genetics. A similar strategy had previously been successfully employed by Niimi et al. to show specific interactions between the *Ca*Cdr1 ectodomain and a D-octapeptide derivative inhibitor [82]. The recovered suppressors for two of such ICL1 mutants (I574A and S593A) mapped to the Q-loop of the C-terminal NBD (R935T) and the Walker A motif (G190R) of the N-terminal NBD, respectively [80] (Figure 3). Biochemical analysis indicated that the compensatory second site mutations helped rebuild coupling interfaces between the NBD and the TMD affected by the alanine substitutions. The same study also identified three suppressors of one of the ICL1 uncoupling mutants, E597A, that mapped to the non-catalytic NBS, reinforcing the latter’s importance in interdomain crosstalk (Figure 3) [80].

The importance of the non-catalytic NBS in the transport cycle of PDR pumps became more evident from two studies by Banerjee et al. [50,58]. They subjected two transport-deficient TMD mutants, V532D and L529A of *Ca*Cdr1, to suppressor studies. The V532D mutant showed an ATPase activity defect while the L529A mutant showed a substrate binding defect [58,59]. Exposure of the *S. cerevisiae* strain expressing *Ca*Cdr1 V532D to ketoconazole led to a second-site mutation W1038S that mapped close to the D-loop of the non-catalytic NBS [58] (Figure 3). Even though transport was recovered in the V532D-W1038S suppressor strain for all efflux substrates tested, the ATPase activity did not improve [58]. The *Ca*Cdr1 homology model showed loss of certain interactions at the level of dimer interface due to the serine substitution [58]. The resultant increased flexibility at the level of NBD dimer due to a lower number of interactions was proposed to modify the interdomain crosstalk and consequently improve transport [58]. On the other hand, the *Ca*Cdr1 L529A mutant yielded the suppressor L529A-Q1005H. In this case, the secondary mutation mapped to the signature sequence of the non-catalytic NBS [50] (Figure 3). Interestingly, the single Q1005H mutant showed increased resistance towards a number of drug substrates while itself being ATPase deficient [50]. It is pertinent to mention that even though the L529A-Q1005H was ATPase competent, it also showed a reduction in the ATPase activity compared to the WT and L529A mutant [50]. These observations suggested the possibility of direct control of the non-catalytic NBS in substrate-translocation via ATP binding [50]. Molecular modeling with the help of available asymmetric transporter structures in ATP bound/unbound states provided further insight [83,84,85]. In the ATP-bound structures of MRP1 and CFTR, a H-bond between the glutamine of the signature sequence and the ribose of the ATP was noted. In contrast, when a histidine is present at the site, as in the case of H1350 in the non-catalytic NBS of CFTR, there is a noticeable displacement of the histidine and the neighbors from the ribose by around 1 to 2 Å, indicating a loose ATP-bound state [50]. Considering that the substrate binding defect manifested by the L529A primary mutant was not corrected in the L529A-Q1005H suppressor protein, there was a need to understand the molecular basis of the functional compensation. Using the ABCG5/G8 based model, it was found that Q1005 along with its proximal region indeed contribute to the closing/opening of the non-catalytic NBS, and as a result, generate the transmission interface with the coupling and connecting helices [50]. The loose ATP-bound state, generated due to the Q1005H substitution, could therefore impact on the conformational changes at the TMDs and result in the restoration of transport in the L529A-Q1005H protein. Similar findings from *Sc*Pdr5 pertaining to the non-catalytic NBS indicate that the non-catalytic NBS indeed plays a primary role in the transport cycle of PDR pumps [56,57]. Substrate selection, a phenomenon which also relies on interdomain crosstalk, has also been shown to be dependent on the NBDs, particularly the H-loop [86]. However, in this particular case, both the canonical and deviant sites appear important [87,88].

Besides providing essential insights into the PDR pump function, the suppressor studies found that a majority of the compensatory mutations did not enhance the ATPase activity, even when the primary defect was in the ATPase machinery, i.e., drug susceptibility was countered by interdomain crosstalk having primacy over an energy intensive step required for reaction cycle completion. The introduction of compensatory mutations, therefore, transformed the efflux pumps into more efficient systems.

Another important level of crosstalk in PDR pumps emerged from a recent study by Tanabe et al., in which the authors screened for FK506 (efflux pump modulator) resistant mutants within *Ca*Cdr1 and *Sc*Pdr5 [89]. Most of the suppressors mapped to either the ECLs or towards the extracellular halves of the TMDs. Moreover, a significant number of the mutations (20) occurred precisely at three *Sc*Pdr5/*Ca*Cdr1 equivalent sites, T550/T540 and T552/S542 of ECL1 and A723/A713 of ECL3, indicating their importance in the phenomenon [89]. With the help of biochemical and structural studies, it was hypothesized that FK506 inhibits efflux by slowing the transporter opening and/or substrate release, while the suppressor mutants modify the critical contact points that facilitate the cotransport of FK506 and other substrates [89].

In comparison to *Ca*Cdr1, interdomain crosstalk has yet to be specifically explored in *Ca*Mdr1. The Prasad group has recently used 58 critical mutants constructed by Redhu et al. for suppressor studies. Notably, the *Ca*Mdr1 G230A mutation, which is located towards the intracellular side within TMH4, yields a suppressor mapping to TMH12 near its extracellular surface. Considering the positions of the primary G230A mutation and the compensatory P528H mutation are in the N-terminal (intracellular side) and C-terminal halves (extracellular side) of the protein, respectively, a novel pattern of interdomain crosstalk emerges. Research is underway to provide a molecular basis for the compensation.

## 6. Challenges Ahead

The advent and subsequent improvement of cryo-electron microscopy technology have assisted the structural resolution of large transporter proteins. This has provided a range of templates that assist in understanding the structure and function of *Ca*Cdr1 and *Ca*Mdr1. As these pumps have remarkable polyspecificity, this feature should be an important consideration in antifungal drug discovery. While alternate antifungals are needed urgently, non-MDR pump substrates should be preferred. Efficient screens that identify MDR pump substrates are available but more extensive use of SAR studies could be of considerable value. There is a compelling need for the rational structure-based design of effective inhibitors that specifically chemosensitize these drug efflux pumps in order to overcome drug resistance in fungal pathogens. Even though some success has been achieved with some of the efflux inhibitors for *Sc*Pdr5, *Ca*Cdr1, and *Ca*Mdr1 [82,90,91], their translation into the clinic has yet to be realized (see reviews on chemosensitizers of antifungal drug efflux by Holmes et al. [11] and Monk and Keniya [92]). There are two primary reasons for this roadblock. The first is the lack of high-resolution structures for yeast PDR pumps. The second is that the data obtained so far using mutagenesis and suppressor genetics have yet to elucidate the mechanisms required for efflux pump inhibition/modulation. These drawbacks are likely to be overcome soon.

## Figures and Tables

**Table 1:** Sequence degeneracy of the residues in the nucleotide binding domains (NBDs) of PDR ABC transporters.

MOTIF	Catalytic (Consensus) NBS ^	Non-Catalytic (Deviant) NBS ^¥^
*Walker A*	GKTT	GCST
*Walker B*	LDE	WDN
*Q-loop*	Q	E
*Signature sequence*	SGG	NVE
*D-loop*	GLD	GLD

^ The catalytic (consensus) nucleotide-binding site (NBS) is made up of Walker A and Walker B motifs and the Q-loop of the C-NBD (pink) and the signature sequence and D-loop of the N-NBD (blue), whereas the ^¥^ non-catalytic (degenerate) NBS is made up of Walker A and Walker B and the Q-loop of the N-NBD and the signature sequence and D-loop of the C-NBD. The degenerate residues in the respective motifs are underlined.

## Data Availability

Not applicable.
